# *Caenorhabditis elegans* larvae undergo early developmental arrest on a diet of Gram-positive bacterium *Enterococcus faecalis*

**DOI:** 10.17912/micropub.biology.000321

**Published:** 2020-10-27

**Authors:** Madhumanti Dasgupta, Nagagireesh Bojanala, Meghana Shashikanth, Varsha Singh

**Affiliations:** 1 Department of Molecular Reproduction, Development and Genetics, Indian Institute of Science, Bangalore, India 560012

**Figure 1. E. faecalis induces early larval arrest in C. elegans:**
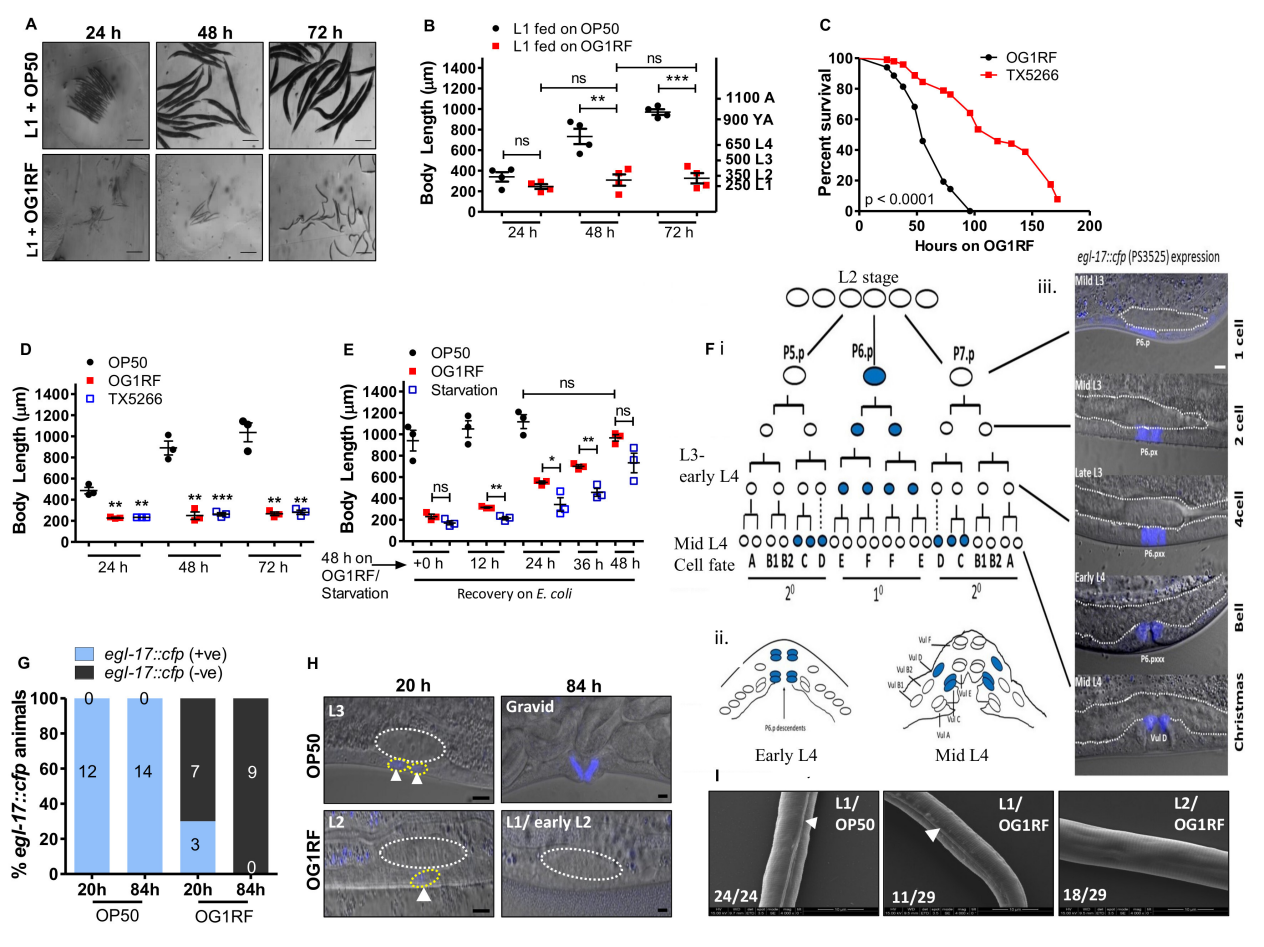
(A) Images of *C. elegans* L1 larvae fed *E. coli* OP50 (Top) or *E. faecalis* OG1RF (Bottom) for 24 h, 48 h and 72 h. Scale bar, 100 μm. (B) Mean ± SEM of body length in populations of L1 larvae fed OP50 or OG1RF for 24 h, 48 h and 72 h. Reported length of L1, L2, L3 and L4 larvae is indicated on the right y axis. (C) Kaplan Meier survival curves for wild type animals fed on OG1RF and Δ*fsrB* *E. faecalis* (TX5266)*.* Also see Table 1. (D) Mean ± SEM of body length in populations of L1 larvae fed OP50, OG1RF or TX5266 for 24 h, 48 h and 72 h. (E) Mean ± SEM of body length in populations of 48 h starved, *E. faecalis*-fed and *E. coli*-fed larvae transferred to *E. coli* plates and measured at regular intervals of 12 hours. n=15-20 animals/ treatment/experiment in panels 1B, 1D and 1E where each point represents the mean of body length of a population of animals (N>3 experiments). *, p less than equal to 0.05; **, p less than equal to 0.01; ***, p less than equal to 0.001; ns-non-significant. In panel D, * indicates significance with respect to OP50-fed animals at the same time point. (F) (i) Cartoon showing vulval invariant cell lineages and morphogenesis in wild-type hermaphrodites (Burdine *et al.*, 1998; Sulston and Horvitz, 1977; Cui and Han, 2003). At larval stage, L3, vulval precursor cells P(5-7).p adopt primary (1°) or secondary (2°) cell fates and undergo invariant cell divisions to produce 22 cells organized into toroids, VulA, vulB1, vulB2, vulC, vulD, vulE, and vulF, shown by A, B1, B2, C, D, E, and F cell types (Sharma-Kishore *et al.*, 1999). Blue coloured oval cells indicate *egl-17::cfp* expression in specific lineages. (ii) Cartoon showing early L4 vulva, when *egl-17::cfp* is exclusively seen in granddaughters of P6.p (1°), and mid-L4 stage, where the expression is shifted to 2° lineage cells, vulC and vulD. (iii) Merged DIC and fluorescence images showing stage specific *egl-17::cfp* in vulval cells. P6.p (1 cell stage/L2), P6.px (2 cell/midL3), P6.pxx (4 cell/mid to late L3), and P6.pxxx (8 cell/early L4 onwards), x denotes one round of mitotic division. Gonads are demarcated with white line. In all animals, anterior is to the left. Scale bar, 5 µm. (G) Expression of *egl-17::cfp* in *C. elegans* L1 larvae fed OP50 or OG1RF for 20 h and 84 h. Duration of feeding on *E. coli* or *E. faecalis* are shown on X-axis and the bars represent % positive or negative *egl-17::cfp* in vulva cells. Numbers on the bars indicate the number of animals which are positive or negative for *egl-17::cfp* expression. Zero on top of blue bars indicate that there are no OP50-fed animals which do not show *egl-17::cfp* expression. (H) Confocal images of *egl*–*17::cfp* in *C. elegans* L1 larvae fed OP50 or OG1RF for 20 h and 84 h. White circles depict gonad primordium and yellow P6.p lineage. In all animals anterior is to the left. Scale bar, 5 µm. (I) Scanning electron microscopy images of larvae fedOP50 for 4 h and fedOG1RF for 72 h. Arrow heads indicate alae. n= 25-30 animals/ treatment. Scale bar, 20 µm.

## Description

*Caenorhabditis elegan*s feeds on bacteria in decomposing vegetation. Lipids, carbohydrates and proteins derived from microbes are digested into fatty acids, simple sugars and amino acids in *C. elegans* alimentary canal and absorbed by intestinal cells containing microvilli. Approximately 80% of fatty acids in *C. elegans* is derived from *E. coli* (Perez and Van Gilst, 2008). Nutrient limiting conditions can cause developmental delay in larvae (Cassada and Russell, 1975; Golden and Riddle, 1982) while complete starvation leads to L1 larval arrest or dauer formation (Baugh, 2013). Interestingly it has been reported that *C. elegans* fed on yeast *Cryptococcus curvatus* show developmental lag (Sanghvi *et al.*, 2016) and growth arrest on Gram-positive bacterium *Enterococcus faecalis* (Garsin *et al.*, 2001). We have recently shown that *E. faecalis* infection causes lipid droplet utilization in adult *C. elegans*, a process termed immunometabolism (Dasgupta *et al.*, 2020). In this study, we have investigated the developmental arrest induced by *E. faecalis* in *C. elegans* larvae to show that the arrest is induced at L1 and L2 larva stage.

To determine the developmental stage at which larvae get arrested upon feeding on *E. faecalis* OG1RF, we exposed a synchronized population of L1 larvae (250 μm body length) to either *E. coli* OP50 or *E. faecalis* OG1RF diet and measured the body length at 24, 48 and 72 hours of feeding on each bacterium (Figure 1A and 1B). We observed that *C. elegans* fed on OG1RF diet were shorter than animals fed on OP50 at all time points (Figure 1B). At 72 hours of feeding, OP50-fed animals had reached adult body lengths measuring >1000 μm. However, OG1RF-fed animals had body length ranging between 250-350 μm, equivalent to L1 and L2 larval stages, indicating an early developmental arrest. Next, we set out to ask if larval arrest on *E. faecalis* diet was due to virulence of *E. faecalis*. The Fsr locus in *E. faecalis* encodes a two-component system (response regulator FsrA*,* peptide lactoneFsrB and histidine kinase FsrC) to control production of virulence factors, gelatinase and serine protease (Qin *et al.*, 2000). *ΔfsrB* *E. faecalis* (TX5266 strain) is attenuated for virulence in *C. elegans* (Figure 1C) as shown earlier (Garsin *et al.*, 2001; Qin *et al.*, 2000). To test if *E. faecalis* virulence was a cause for arrest, we fed L1 larvae with TX5266 strain and found that it caused larval arrest similar to the arrest caused by OG1RF feeding (Figure 1D). This indicated that factors other than pathogenesis of *E. faecalis* are likely responsible for developmental arrest in *C. elegans*. To understand if *E. faecalis*-induced larval arrest was reversible, we allowed 48 h starved larvae, 48 h OG1RF-fed larvae and 48 h OP50-fed larvae to resume/continue feeding on OP50 for additional 12, 24, 36 and 48 hours (Figure 1E). As shown, both starved and *E. faecalis* arrested larvae resumed larval development when fed *E. coli* diet, although starved larvae were slower in resuming development.

To further confirm that *E. faecalis* induced arrest during larval development, we also studied proliferation and lineage progression in hermaphrodite vulva (Figure 1F-1H). Development of vulva is tightly synchronized with larval development. In *C. elegans* hermaphrodite, vulva development from vulval precursor cells (VPC) is dependent on signalling from the gonadal cell called the Anchor Cell (AC). AC secretes LIN-3/EGF (Epidermal Growth Factor) to induce vulva fate in VPCs- P5.p, P6.p and P7.p- of the equipotent P(3-6).p lineage cells. P6.p, the VPC closest to anchor cell adopts 1^0^ cell fate, and VPCs P5.p/P7.p adopt 2^0^ fates. The EGL-17/FGF (Fibroblast Growth Factor) is induced transcriptionally by inductive and notch signalling in vulva cells (Burdine *et al.*, 1998; Sulston and Horvitz, 1977). From early L3 to late L3, EGL-17 is expressed in P6.p/1^0^ lineages and at mid L4 it switches to 2^0^ lineage cells, VulC and VulD (Figure 1F). We followed P6p fate in OP50 and OG1RF-fed larvae. At 20 hour post-feeding (time prior to VPC P6.p division), 100% of OP50-fed larvae showed positive *egl-17*::*cfp* expression corresponding to L3 larval stage. However, in OG1RF-fed larvae only 30% larvae showed weak expression in P6.p cell corresponding to late L2 stage while 70% had no GFP expression indicating an L1 or early L2 stage. At 84 hours of feeding, we observed *egl-17*::*cfp* expression in vulD corresponding to adult stage in OP50-fed animals, but we observed no cell division in P6.p lineage in OG1RF*–*fed larvae indicating arrest at L1 or early L2 stages (Figure 1G and 1H). These observations confirmed that the vulva development in OG1RF-fed larvae was indeed arrested at early larval stages as VPCs did not divide and never acquired vulva fates. For the final confirmation, we also examined OG1RF arrested larvae for the presence of alae, longitudinal ridges in the cuticle which are observed only in L1, dauer and adult stage of *C. elegans* (Cox *et al.*, 1981). We found that 72h-fed OG1RF larvae comprised of a mixed population of L1s and L2s, with 38% (11 out of 29) animals showing alae (L1) and the remaining 62% (18 out of 29) did not have alae (L2) (Figure 1I).

Taken together, our study shows that *E. faecalis* OG1RF cocci diet poorly supports growth of *C. elegans* larvae and cause development arrest at early larval stages.

## Methods

**Nematode maintenance**

*C. elegans* were maintained at 20°C on nematode growth medium (NGM) seeded with OP50 *E. coli*. Wild type N2 was obtained from *Caenorhabditis* Genetics Center, Minnesota, USA. Strains used in this study have been listed in Table 2 (mentioned in the reagents section). All feeding assays were conducted at 25˚C.

**Bacterial strains and Growth media**

Bacterial strains used in this study are listed in Table 2. *E. coli* OP50was grown in LB at 37˚C for 8 hours and seeded on NGM plates containing streptomycin. *E. faecalis* strains were grown in BHI broth with appropriate antibiotics for 5 hours at 37˚C and 50 μl culture was spread onBHI agar plates with respective antibiotics and kept at 37˚C overnight.Gentamycin (50 μg/ml) was used for *E. faecalis* OG1RF and Rifampicin (100 μg/ml) was used for TX5266 strain (Table 2).

**L1 synchronization**

NGM agar petri plates with 150 gravid adults each, were prepared for bleaching (2-3 petri plates for body length measurement and 10-12 petri plates for SEM experiment). Animals were washed with phosphate buffer saline (PBS) in a 15ml tube, after which a 1:1 mixture of 2X Bleach solution and PBS was added. Bleach solution was prepared by adding 900 μl sodium hypochlorite solution, 700 μl distilled water and 400 μl of 5N NaOH. The tube was shaken vigorously for 1-2 minutes until eggs were released into the solution. The eggs were immediately washed with 10 ml PBS and collected by centrifugation at 3000 rpm for 2 minutes. This step was repeated 5 times, following which eggs were resuspended in 5 ml PBS and maintained on a rotator at room temperature, for 20 hours for eggs to hatch to L1 larvae. L1s were spotted on *E. coli* and *E. faecalis* lawns, for carrying out further experiments.

**Body length measurements**

Synchronized L1 larvae (Figure 1A, B and D) were spotted on *E. coli* and *E. faecalis* lawns. Every 24 hours, 15-20 animals/time point/diet were mounted on 2% agarose pad and imaged using AxioCam I Cm1, Zeiss microscope. Body length of animals was measured using Image J software. Each experiment was repeated at least three times. For larval rescue experiment, L1s were spotted on *E. coli*, *E. faecalis* lawns or on plate NGM agar plates (for starvation) for 48 hours, after which they were transferred back to *E. coli* lawn and imaged after every 12 hours.

**Vulva Development study**

PS3525 strain (*egl-17::cfp + unc-119(+))* was used to trace vulval precursor cell lineage during development. Synchronized L1s of PS3525 strain were exposed to *E. coli* and *E. faecalis* and imaged at 20 h and 84 h post exposure using LSM880 airyscan microscope. The number of vulva cells showing *egl-17::cfp* fluorescence were counted. 10-20 animals were used for each time point for each diet. Vulval precursor cell lineage is described (Figure 1, F-H.).

***C. elegans* survival assay**

To assess the survival of *C. elegans* during infection, we performed survival assays. A single *E. faecalis* bacterialcolony was inoculated in 2 ml of BHI broth. 50 μl of bacterial culture was spread on 60 mm BHI agar plates with appropriate antibiotic and incubated at 37˚C overnight. 100-120 synchronized young adult animals were exposed to *E. faecalis* at 25°C and scored for survival at the times indicated in Figure 1C. Animals were considered dead when they failed to respond to touch. Each survival assay was performed three times.

**Scanning Electron Microscopy**

Synchronized L1s exposed to *E. faecalis* lawn for 72 hours and *E. coli* lawn for 3-4 hours, were washed with phosphate buffer saline (PBS) three times and collected. These animals were incubated in fixation buffer (5% glutaraldehyde, 5% formaldehyde and 0.2M HEPES, pH 7.3) overnight at room temperature. Next day animals were postfixed in a solution containing 2% osmium tetroxide, 200mM CaCl_2_ and 12.5% K_3_[Fe(CN)_6_] for 4 hours at room temperature. Animals were washed with sodium phosphate buffer three times, followed by four washes with water. Next, the animals were dehydrated through increasing concentration of ethanol and finally suspended in 100% ethanol overnight. This protocol was modified from (Shemer *et al.*, 2004). The following day, animals were spotted on a coverslip, coated with gold and imaged using FEI XL-30 ESEM scanning electron microscope at Indian Institute of Science Advanced Microscopy Facility.

**Statistics**

Statistical analysis was done using Graphpad Prism software. Unpaired t-test was used for comparison of mean body lengths obtained from measurements across individual experiments (N) on populations composed of 15-20 individuals (n). Kaplan-Meier analysis was performed to calculate survival fractions and the Log Rank test to compare survival curves (see Table 1). TD_50_, the time to death for 50% of the population was calculated as described (Singh and Aballay, 2006). Survival curves were considered different from the appropriate control when P values were < 0.05.

## Reagents

Table 1. **Statistical analysis of Kaplan Meier Survival Curves.**

**Table:** 

**Pathogen Used**	**Trial no**	**Strain Genotype**	**TD_50_** **(Hours)**	**No. of animals used / No. of animals censored**	**Statistical significance**
*E. faecalis*	I	WT OG1RF WT *ΔfsrB* OG1RF	54 110	100/32 104/40	P < 0.0001
*E. faecalis*	II	WT OG1RF WT *ΔfsrB* OG1RF	51 74	100/36 100/49	P < 0.0001
*E. faecalis*	III	WT OG1RF WT *ΔfsrB* OG1RF	52 86	100/24 100/21	P < 0.0001
*E. faecalis*	IV	WT OG1RF WT *ΔfsrB* OG1RF	44 98	100/41 100/55	P < 0.0001

Table 2. **List of strains used in this study.**

**Table:** 

S. no.	Strain name	Source
1.	*Caenorhabditis elegans* N2 (Bristol)	*Caenorhabditis* Genetics Center (CGC), University of Minnesota
2.	*C. elegans* PS3525 *egl-17::cfp + unc-119*(+)	*Caenorhabditis* Genetics Center (CGC), University of Minnesota
3.	*E. coli* OP50. Uracil auxotroph. *E. coli B*	A gift from Dr. Alejandro Aballay, Oregon Health and Science University (OSHU)
4.	*E. faecalis* OG1RF	A gift from Dr. Alejandro Aballay, OSHU
5.	*E. faecalis* TX5266 Δ*fsrB* OG1RF	A gift from Dr. Barbara Murray, McGovern Medical School, UT Health
